# Paediatric Spinal Cord Injury: A Review of Current Management

**DOI:** 10.7759/cureus.63306

**Published:** 2024-06-27

**Authors:** Chikolum Ejide, Soham Bandyopadhyay, Kokila Lakhoo

**Affiliations:** 1 Oxford University Global Surgery Group, University of Oxford, Oxford, GBR

**Keywords:** spine surgery, orthopedics, neurosurgery, pediatrics, emergency medicine, trauma, spine injuries, spinal cord injury, pediatric spine

## Abstract

Spinal cord injury (SCI) in the paediatric population is considered a separate entity from the same injury in adults due to the unique anatomical, physiological, and biomechanical properties of the pediatric spine. No comprehensive, standardized, international guidelines currently exist for physicians to follow regarding the management of paediatric spinal cord injuries. Therefore, a narrative literature review approach was employed to explore the management of paediatric spinal cord injuries. The review adhered to the methodological frameworks that entailed identifying a curated selection of pertinent articles on the topic, rather than an exhaustive comprehensive search that is utilised in systematic reviews, this was followed by a reflective interpretation of their content. Using the electronic databases, PubMed and Google Scholar, a search of peer-reviewed studies conducted only in the English language was included. Only studies in which the full article was available were included. Paediatric populations are defined as individuals aged between 0 and 18 years. In total, 26 studies were included in our review.

We conclude that it is necessary to factor in specific paediatric considerations, such as disproportionate head size, increased ligament laxity, increased prevalence of upper cervical injury, and future development of scoliosis, in the prehospital, medical, and surgical management of paediatric spinal cord injuries. Clinicians should be made aware of these considerations, as they can improve the outcomes in the paediatric population who suffer from this devastating injury. There is a lack of high-quality studies and data concerning the paediatric population who have sustained SCIs. This literature review highlights the available data and calls for more studies to be conducted in this field.

## Introduction and background

Spinal cord injury (SCI) results from injury to the neurologic structures of the spinal column and can be a profoundly devastating event. It can cause severe impairments that can impact almost every facet of an individual’s life. SCI incidence is relatively low as compared with other chronic diseases; however, the consequences are disproportionally high. In addition to the physical and psychosocial trauma, the economic burden is substantial and the consequential financial and healthcare costs may also have severe impacts on society and the individual [1].

SCI has a bimodal distribution, with high and low energy mechanisms affecting the younger and older populations, respectively. A young person, having been a previously healthy individual, may abruptly find themselves reliant on lifelong medical, surgical, and rehabilitative care and support. The most common aetiologies of SCI are road traffic accidents (RTAs), violence, sporting incidents and falls in the older population [2].

While it can be devastating at any age, SCI can present distinct challenges in the paediatric population. The unique anatomy, physiology and biomechanics of the paediatric spine lead to injury patterns distinct from those seen in adults [3]. Despite the apparent necessity for specialised management, there exists a noticeable scarcity of dedicated research and established protocols for the effective management of paediatric SCI. Present guidelines derive from research focused on the adult population, underscoring the critical need for the development and implementation of paediatric-specific SCI protocols across diverse nations and healthcare centres. This literature review aims to address the gaps in standardised research and protocols for paediatric SCI by exploring various facets of its management.

Paediatric spine considerations

**Figure 1:**
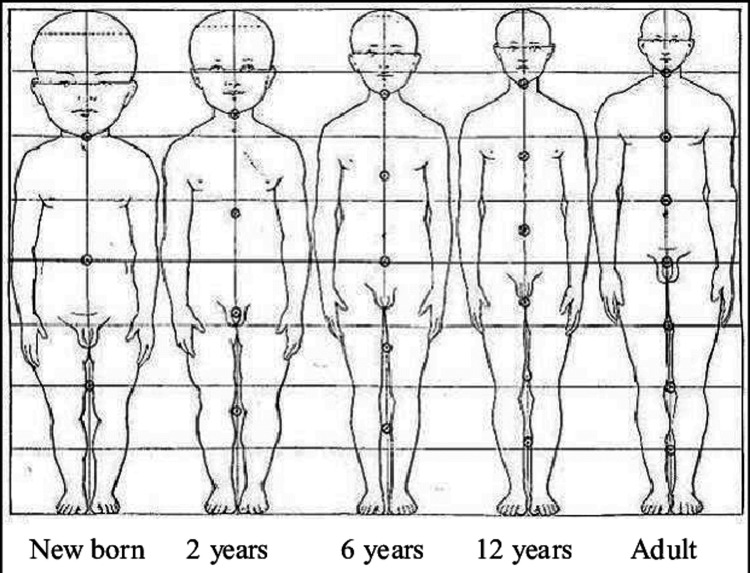
Proportions of the body from a newborn baby until adulthood Image credit: Donald Huelke, 1992 [4]

The younger paediatric population, especially before the age of eight years, have heads that are disproportionately large relative to their bodies, as well as underdeveloped neck musculature (Figure 1). This means that the occipitocervical region is more vulnerable to injury, leading to a higher prevalence of upper cervical injuries in this population [3].

## Review

Prehospital management

Appropriate prehospital management can improve the neurological status of patients upon hospital arrival, a credit often attributed to the immobilisation of the cervical spine during initial response following the Advanced Trauma Life Support (ATLS) protocol [5]. Improved neurological outcomes can also be attributed to resuscitation strategies that serve to improve perfusion to the spinal cord. Research suggests that as much as 25% of SCIs occur post-primary injury, wherein pathological movement of the injured spine induces additional mechanical trauma [6]. Consequently, the implementation of immobilisation measures to forestall further spinal cord damage has yielded improved outcomes, with three-point immobilisation of the cervical spine recognised as the gold standard [7].

Paediatric considerations necessitate a more nuanced approach to immobilisation. Children under 10 years old, owing to their disproportionately larger heads in relation to their bodies, experience neck flexion on a standard spine board. Placing them in a supine position may induce cervical kyphosis, potentially exacerbating injuries to the cervical spine [7]. This underscores the importance of using specifically adapted paediatric spinal boards that allow for a neutral spine position. In the absence of such equipment, maintaining a neutral position of the spine with blocks or rolled-up towels on either side of the head, secured with tape, aligns with advanced paediatric life support principles and the National Institute of Health and Care and Excellence (NICE) guidelines [8].

Given the association of spinal injuries with head trauma and other severe injuries, unconscious patients, especially those with a Glasgow Coma Scale (GCS) of 8 or less, may necessitate emergent intubation. Respiratory complications are a leading cause of morbidity and mortality in the general population with acute SCI, occurring in 36-83% of cases [9]. The paediatric population, particularly susceptible to cervical spine injuries, may face ventilatory compromise due to damage to C3-C5 diaphragmatic innervation. Injuries at or above this level result in apnoea and require ventilatory support [9,10]. Intubation may become imperative, which emphasises the critical importance of adherence to ATLS protocols by initial responders and the availability of paediatric-appropriate airway and ventilation equipment at the scene.

Hospital management

Imaging

The diagnosis of SCI relies on a combination of clinical evaluation and radiographic assessment. This holds particular significance in the paediatric population due to potential challenges in verbal communication of symptoms and the potentially subtle nature of diagnostic imaging findings in children. When available, scans should be reported by a radiologist experienced in imaging the paediatric spine, given the distinct characteristics of the growing spine [11]. For example, the paediatric spine has facet joints that are more horizontal, ligaments and joints that are more lax and vertical bodies that are wedge-shaped. These differences can also lead to pseudosubluxation of C2 on C3 in which there appears to be a forward slippage of one vertebral body on another [12].

While CT scans offer high specificity for detecting cervical spine injuries, apprehensions about ionizing radiation and its long-term risks in children often make physicians cautious. Level I evidence on which children necessitate a CT scan is limited, with most recommendations grounded in level II-IV evidence. The National Institute for Health and Care Excellence (NICE) recommends a CT scan within one hour when specific risk factors are present, including focal neurological signs, paraesthesia in the upper/lower limbs, GCS < 13 on admission, intubation, when a definitive diagnosis of cervical spinal injury is needed prior to manipulation in surgery or anaesthesia, clinical suspicion of spinal injury despite normal radiographs, difficulty in radiography or identification of significant bony injury [13].

Spinal cord injury without radiographic abnormality (SCIWORA) is a phenomenon found predominantly in the paediatric population sustaining SCI. Up to 60% of children and adolescents sustaining an SCI may have no evidence of radiographic abnormalities on plain X-rays and CT [14]. It likely occurs due to the inherent elasticity and ligamentous laxity of the paediatric spine. These unique biomechanics allow for the deformation of musculoskeletal structures beyond physiologic limits, causing direct spinal cord trauma followed by a spontaneous reduction of the bony spine [14].

The prevalence of SCIWORA in the paediatric population underscores the critical choice and availability of appropriate imaging. Pang et al. performed a study on 55 children with SCIWORA, revealing that 15 cases experienced delayed onset of neurological abnormalities, often accompanied by subtle transient warning symptoms such as paraesthesia or subjective weakness. The delay ranged from 30 minutes to 96 hours before deterioration onset. Eight other children suffered a second SCIWORA 3 days to 10 weeks after the initial event [15]. The intricacies of SCIWORA, delayed onset of neurological abnormalities and communication challenges in younger children contribute to the complexity of the initial diagnosis of paediatric SCI. In cases where children exhibit neurological spinal cord signs without X-ray or CT abnormalities, a magnetic resonance imaging scan (MRI) is recommended [16]. It has been shown that MRI can be used to predict early neurologic outcomes [16].

Methylprednisolone

The utilisation of high-dose steroids in the management of spinal cord injury remains a subject of controversy, with its role in the paediatric population particularly elusive due to a scarcity of relevant literature. In the adult demographic, the National Acute Spinal Cord Injury Study (NASCIS) II and III trials are frequently referenced to advocate for high-dose methylprednisolone as an effective intervention for acute spinal cord injuries [17]. However, these trials were conducted in 1996 and have since sparked controversy, primarily stemming from concerns regarding statistical analysis and the overall clinical benefit, prompting some clinicians to question the justification of this approach. Notably, the administration of high-dose steroids is associated with a significant increase in infection risks, potentially leading to prolonged hospital stays and dependence on ventilators. In a more recent meta-analysis, the authors recommended against the use of high-dose steroids in the event of acute SCI [18].

Pettiford et al. conducted a comprehensive review of existing literature on the role of high-dose methylprednisolone in paediatric SCI. Their findings suggest that the use of steroids in paediatric patients is linked to increased infection risks without accompanying neurological improvements [19]. The dearth of studies in this population highlights the need for randomized controlled trials to ascertain the potential benefits of steroid use following SCI in children. Despite this, the 2019 acute spinal cord injury recommendations from the World Federation of Neurosurgical Societies (WFNS) cautiously suggest a 24-hour infusion of high-dose methylprednisolone within eight hours of injury in selected young patients with acute SCI [20]. The guidelines further emphasize the lack of high-evidence pharmacological agents for acute SCI. Overall, because of the lack of randomised controlled trials in this population, there is a lack of strong evidence to definitively conclude the benefit of high-dose steroids in the paediatric population.

Surgical management

External Fixation

Ensuring the immobilisation of the spine to mitigate secondary injury is a crucial element in the care of suspected or confirmed spinal cord injuries. The application of a Halo device, an external fixation method, represents a nonoperative approach to spinal trauma and deformity. This technique employs longitudinal force to stabilise the spine, restore alignment and indirectly decompress the spinal cord [21]. Spinal traction, whether used independently or as an interim measure before definitive surgical stabilisation, can be effective, particularly for reducing fracture-dislocations in the cervical spine.

In paediatric cases, careful consideration is imperative when employing the Halo ring due to the heightened risk of complications. Limpaphayom et al. evaluated the complications of halo use in 68 children and found the complication rate to be significant at 53% [21]. These complications include pin site infections, skull perforation, brain abscess formation, bradycardia, cranial nerve injury, pneumocranium, and logistical issues such as over-distraction injuries. Specialised paediatric halo rings with 8-10 pins are utilised, and in children under the age of six, a CT head is performed to identify areas of adequate skull thickness, considering the considerable variability in thickness below this age [22].

Paediatric patients are also especially susceptible to atlanto-occipital dislocation (AOD), given that increased ligament laxity and traction could exacerbate neurological injuries. Therefore, early recognition of AOD before attempting halo fixation is crucial [23].

Operative Management

In certain scenarios, internal fixation may be indicated to prevent the spinal cord from further harm and provide stability. Eleraky et al. retrospectively reported on 102 cases of paediatric cervical spine injury and further reviewed the available literature on the management of paediatric SCI to compare it to their own experience. Surgical fixation becomes necessary for unstable injuries, progressive deformities, non-reducible deformities, and to decompress neural structures [24]. Some experts advocate for surgical decompression within 24 hours, or even 8 hours, as the most efficient strategy for preserving function and facilitating recovery [25]. However, definitive conclusions should be tempered until additional randomised controlled trials are conducted.

Long-term paediatric considerations arise due to the growing spine, as surgical instrumentation may impede growth and predispose the paediatric spine to scoliosis development. Research indicates that the risk of developing scoliosis following spinal cord injury in younger children before their growth spurt is almost inevitable; Mulcahey et al. determined that of 217 subjects included in their study, with an average age of 13.2 years at follow-up, 100% of them went on to develop neuromuscular scoliosis after an average of 4.2 years post-injury [26]. Long-term physical therapy and ongoing follow-up are essential components of the comprehensive care required for these patients.

## Conclusions

SCI presents a formidable challenge; its multifaceted nature, marked by primary and secondary insults, necessitates a comprehensive understanding and multidisciplinary approach to management. SCI impacts individuals of all ages; however, there are unique challenges and considerations in the paediatric population. These unique characteristics make SCI in the adult population a separate entity from the spinal injuries seen in the paediatric population. The paucity of research in the management of this devastating condition has contributed to the absence of international standardised guidelines in the management of SCI in this population.

Prehospital management, encompassing effective immobilisation and adherence to ATLS protocols, plays a pivotal role in optimising outcomes. In the young child, care must be made to account for their disproportionately larger heads when attempting to keep their spine in a neutral position. The evolving understanding of the role of high-dose methylprednisolone in SCI, particularly in children, necessitates cautious consideration, and given the associated risks and limited evidence, this should be an informed decision based on the individual patient and the managing clinician. Surgical management, ranging from external fixation with halo devices to internal fixation, represents a critical aspect of SCI care. Paediatric considerations in surgical interventions, acknowledging the impact on spinal growth and the predisposition to subsequent scoliosis, add another layer of complexity. This literature review highlights the available data and calls for more studies to be conducted in this field.
